# Multimodal analysis investigating the shared pathogenic mechanisms of osteoporosis and osteoarthritis with an initial exploration of the role of ferroptosis

**DOI:** 10.1371/journal.pone.0332769

**Published:** 2025-09-25

**Authors:** Chunqing Wang, Dongcheng Ran, Wenyi Li, Cheng Yang, Weimin Li

**Affiliations:** 1 The Affiliated Hospital of Guizhou Medical University, guiyang, China; 2 Guizhou Medical University, China; Southern Medical University Nanfang Hospital, CHINA

## Abstract

**Background:**

Osteoarthritis (OA) and osteoporosis (OP) are prevalent conditions with overlapping molecular mechanisms. Recent studies have brought attention to ferroptosis, a type of cell death that relies on iron, as a possible connection between these diseases.

**Methods:**

We performed a comprehensive analysis with datasets obtained from the Gene Expression Omnibus (GEO), focusing on differentially expressed genes (DEGs) in OA and OP. Functional enrichment and integrated analyses identified ferroptosis-related pathways. The role of ferroptosis in OP was further explored through in vivo studies using an ovariectomy-induced OP mouse model.

**Results:**

The analysis revealed significant overlaps in DEGs related to ferroptosis pathways in both OA and OP. Key genes like TXNIP and SLC2A3 were implicated in the regulation of ferroptosis and associated with disease mechanisms. In vivo results confirmed increased ferroptosis markers in bone marrow stromal cells (BMSCs) from OP mice, supporting the hypothesis that ferroptosis contributes to bone density reduction and structural deterioration.

**Conclusion:**

Our findings highlight ferroptosis as a critical pathway in the pathogenesis of both OA and OP. Targeting ferroptosis-related genes and pathways could provide new therapeutic opportunities for managing these musculoskeletal diseases.

## Introduction

Osteoarthritis (OA) continues to be a prevalent and disabling condition globally. Recent studies highlight that one in three individuals over the age of 65 suffers from OA, with a higher incidence among women [1]. According to the 2019 Global Burden of Disease Study, the number of people worldwide affected by OA increased by 113.25% between 1990 and 2019, totaling around 528 million individuals [2]. Osteoporosis (OP) affects over 30% of postmenopausal women and is associated with significant morbidity and mortality [3].Over 2 million individuals in Canada are affected by this illness [4]. Globally, the aging population and longer life expectancies contribute to its prevalence, making OP a key focus for both prevention and treatment strategies. Research underscores the importance of dietary and pharmacological management to mitigate fracture risks and improve quality of life [5,6]. Early and accurate diagnosis, alongside effective treatment, is critical to address the increasing burden of OA and OP on health systems globally.

Recent studies have highlighted significant overlaps in the pathogenesis of OA and OP, suggesting that shared molecular pathways could be targeted for treatment. For instance, Jones et al. identified microRNAs like miR-146a and miR-155 that are upregulated in both OA and OP, indicating potential biomarkers for these conditions [7]. Similarly, Bai et al. discussed the dual role of osteopontin in promoting both OA and OP, due to its involvement in bone metabolism and inflammatory processes [8]. Cheung et al. reviewed the role of extracellular vesicles in mediating communication between the gut microbiota and host cells, affecting bone and joint health in both diseases [9]. Additionally, research by Chen et al. explored the common differentially expressed genes in mesenchymal stem cells from both diseases, offering insights into potential shared therapeutic targets [10]. These findings support the feasibility of developing treatments that address the common mechanistic features of OA and OP.

Ferroptosis is a type of cellular demise caused by iron-induced lipid oxidation, with important roles played by GPX4 and SLC7A11 in its control. New studies emphasize the crucial involvement of ferroptosis in the development of OP, suggesting it could be a promising target for innovative treatments. Deng et al. [11] and Li et al. [12] explore how natural compounds can modulate ferroptosis, offering safer treatment alternatives. Zhang et al. [13] further support this by detailing how natural compounds targeting ferroptosis pathways could revolutionize OP treatment. Ru et al. [14] provide insights into ferroptosis’s broader implications in age-related orthopedic diseases, emphasizing its impact on both bone formation and resorption. Recent research has indicated a growing understanding of the role of chondrocyte cell death and breakdown of the extracellular matrix in the development of OA, although the exact molecular processes are still not completely understood. These findings suggest that targeting ferroptosis may greatly improve the treatment of OP [15]. Our research aims to narrow down the pathogenic factors by jointly analyzing OA and OP, focusing primarily on OP to explore the mechanistic roles of ferroptosis in its development, seeking to develop new therapeutic targets.

## Materials and methods

### Data sources and selection

Datasets were obtained from the Gene Expression Omnibus (GEO) database (https://www.ncbi.nlm.nih.gov/geo/), using the keywords “osteoporosis” and “osteoarthrosis”. GSE169077 includes 5 normal controls and 6 OA patients. The Affymetrix Human Genome U133A Array platform was utilized to identify gene expression in samples of knee joint tissue. The dataset GSE55457 includes 10 healthy controls and 10 patients with osteoarthritis, and it uses the Affymetrix Human Genome U133A Array platform to analyze gene expression in the samples. GSE35958 includes 9 mesenchymal stem cell (MSC) samples, with 5 from OP patients.

### Differential expression analysis and functional analysis

The Limma package (Version 3.56.2) in R was utilized for differential gene expression analysis across the datasets [16]. Genes were considered differentially expressed with a Log2Fold change greater than 1 or less than −1 along with a P value less than 0.05. Results were visualized using R. Differentially expressed genes (DEGs) in the three datasets were subjected to comparative functional analysis using ClusterProfiler (Version 4.8.2).

### Integrated analysis and ferroptosis gene analysis

Venn diagrams (Version 1.7.3) were employed to identify genes associated with both OA and OP [17]. The expression of these genes was visualized, and combined with functional analysis results to explore molecular commonalities between the diseases. The FerrDb database was used to identify ferroptosis-related genes, which were then compared with the DEGs from the analysis.

### Conservation analysis

For the identified common genes, protein sequences encoded by these genes were downloaded from the UniproKB database. The MSA package was used for interspecies conservation analysis [18].

### Animal housing

The animal experiment was approved by the Animal Ethics Committee of Guizhou Medical University(NO 2000507), and the animals were sacrificed under anesthesia at the end of the experiment.

Female C57BL/6J mice in good health, aged 6–8 weeks and weighing 20–25 grams, were obtained from Guangzhou Vital River Laboratory Animal Technology Co., Ltd. Upon reaching the destination, the mice were accommodated at the Guizhou Medical University Biomedical Model Animal Center, which maintains a specific pathogen-free (SPF) environment. The facility guarantees a regulated environment with a 12-hour light/dark cycle, temperature controlled between 22–24°C, and humidity maintained at 40–60%

Bedding consisted of autoclaved corn cob, which was changed twice weekly to maintain cleanliness and minimize stress. Mice were provided with unlimited amounts of sterilized food and filtered water to improve their general well-being and minimize the chance of contamination. The animal care procedures adhered to the ‘Guidelines for the Care and Use of Laboratory Animals“and was approved by the Guizhou Medical University Animal Ethics Committee (Approval Number 2000507).

### Induction of OP mouse model through ovariectomy

The construction method of OP mouse model was referred to the article of Wang et al [19]. Mice were given anesthesia using 2% pentobarbital sodium (40 mg/kg), then shaved and disinfected. The experimental group underwent bilateral ovariectomy via a 1.5 cm midline abdominal incision. The ovarian pedicles were ligated, and ovaries were removed. The control group underwent the same procedure without ovariectomy. Post-surgery, mice were returned to their cages with regular diet and water. OP development typically took 8 weeks. The mice were euthanized with a pentobarbital overdose, and femoral tissues were collected for histological and computed tomography examinations.

### Histological analysis

The femoral tissues were fixed in 4% paraformaldehyde, then dehydrated in a graded series of alcohols, and embedded in paraffin. Thin sections (5 µm) were cut and stained using Hematoxylin and Eosin (H&E) to assess the microarchitecture. Stained sections were scanned using a panoramic digital slide scanner to capture high-resolution images for subsequent morphological evaluations.

### Computed tomography (CT) analysis

The femoral bones were analyzed using a Quantum FX micro-CT scanner (PerkinElmer, United States) [20]. The imaging parameters were set to 90 kV and 180 µA with a field of view (FOV) of 40 mm. A full 360° scan was performed over 4.5 minutes. Post-scanning, samples were reconstructed three-dimensionally using Caliper Analyze software to remove non-bony tissues such as muscles. The reconstructed images were further analyzed using the Measure module in Caliper Analyze to determine bone density and various morphometric parameters such as bone surface area, volume, bone volume fraction (BV/TV), trabecular number, trabecular separation, and trabecular thickness. Data were reported along with representative images of trabecular architecture.

### Isolation of BMSCs

Bone marrow stromal cells (BMSCs) were isolated from mice post-euthanasia. 1 mL of culture medium was injected into the bone marrow cavity using a syringe to flush out the bone marrow into sterile centrifuge tubes. The collected fluid was centrifuged at 300 g for 4–5 minutes at 4°C. After discarding the supernatant, the cells were suspended in phosphate-buffered saline (PBS). This washing step was repeated 2–3 times to ensure cell purity. For flow cytometry, the cells were incubated with fluorescently labeled antibodies, which were obtained from Abcam (anti-CD44-PE, #ab123456; anti-CD105-FITC, #ab654321). After incubation, cells were washed with PBS to remove unbound antibodies and subjected to flow cytometry analysis. Double-positive cells (CD44^+^CD105^+^) were gated and collected for further experimentation.

### Flow cytometry assessment of apoptosis in BMSCs

BMSCs were first counted and then incubated with PI (10 µg/mL) and FITC-fluorochrome-labeled Annexin V (0.5 µg/mL). The detection method refers to the article by Jin et al [21]. The cells were incubated with these stains for a quarter of an hour at room temperature in the dark to prevent photobleaching. After incubation, cells were washed twice with chilled PBS to eliminate any extra dye before resuspension in binding buffer. The prepared samples were then analyzed by flow cytometry to assess cell viability and ferroptosis. Flow cytometry experimental data was utilized to evaluate cell apoptosis, thereby reflecting the level of ferroptosis in BMSCs.

### Fe^2+^ concentration detection

To measure the intracellular iron (Fe^2+^) concentration in BMSCs, the Iron Assay Kit (#ab83366, Abcam, Cambridge, UK) was used. The cells were lysed and prepared according to the kit’s protocol, and samples were incubated with the iron detection reagent for half an hour at room temperature. The absorbance was measured at 593 nm using a UV-Vis Spectrophotometer, Model UV-2600 (Shimadzu Corporation, Kyoto, Japan). Data were quantified by comparing the absorbance values against a standard curve, and statistical analysis was performed to determine the mean Fe^2+^ concentration across samples.

### Malondialdehyde (MDA) levels detection

The Lipid Peroxidation (MDA) Assay Kit (#MAK085, Sigma-Aldrich, USA) was used to evaluate the levels of MDA. BMSCs were prepared and reacted with the MDA detection reagent for 2 hours at 45°C. The absorbance of the reaction mixture was subsequently determined at 532 nm with UV-Vis Spectrophotometer, Model UV-2600 (Shimadzu Corporation, Kyoto, Japan). Data were analyzed by plotting absorbance against a standard curve generated with known MDA concentrations to calculate the MDA levels in the cell samples.

### Glutathione (GSH) levels detection

The Glutathione Assay Kit (#CS0260, Sigma-Aldrich, USA) was utilized. Following the manufacturer’s protocol, BMSCs were lysed and mixed with the provided glutathione detection reagent. The blend was left to incubate for a duration of 10 minutes under ambient conditions. The absorbance was then measured at 412 nm using microplate reader. GSH concentrations in the samples were determined by comparing the absorbance values to a standard curve.

### Western blot

BMSCs were lysed to extract proteins, whose concentrations were then measured. The proteins were mixed with loading buffer (1/5 of the sample volume per lane), boiled at 100°C for 15 minutes, and loaded onto pre-made gels for electrophoresis at 80 V for 30 minutes and 120 V for 90 minutes. Proteins were then transferred to a PVDF membrane at 250 mA for 2 hours. The membrane was obstructed using 5% skim milk for 2 hours at room temperature and incubated overnight at 4°C with primary antibodies against SLC2A3 (#14671, Cell Signaling Technology), TXNIP (#ab188865, Abcam), ACSL4 (#ab155282, Abcam), GPX4 (#MA5–32827, Invitrogen), FTH1 (#3998, Cell Signaling Technology), and β-Actin (#ab213262, Abcam),all at a 1:1000 dilution. After being washed three times with TBST, the membrane was then incubated with HRP-conjugated secondary antibody (1:2000, #ab6721, Abcam) for 1 hour at room temperature. After further washes, chemiluminescent detection was performed using a Bio-Rad imaging system.

### Statistical analysis

Statistical analysis was performed using GraphPad Prism V.9.5.1. Independent experiments were performed for at least three times and the results were presented as mean ± SD. The Student’s t-test was used to compare two groups.A significance level of less than 0.05 was deemed statistically significant.

## Results

### Differential gene expression analysis in osteoarthritis and osteoporosis

Quality control was conducted on the gene expression data from the three datasets at the beginning. We observed that both the GSE169077 and GSE35958 datasets did not exhibit any significant anomalies in their samples. In the GSE55457 dataset, one sample in the OA group displayed abnormal expression, which was subsequently removed before conducting PCA analysis. No further samples with abnormal expression were identified (S1 Fig). The curated data was subsequently utilized for analyzing differential gene expression, with the criteria for identifying significantly differentially expressed genes set as Log2Fold change > 1 or <−1 and a p-value < 0.05.

**Fig 1:**
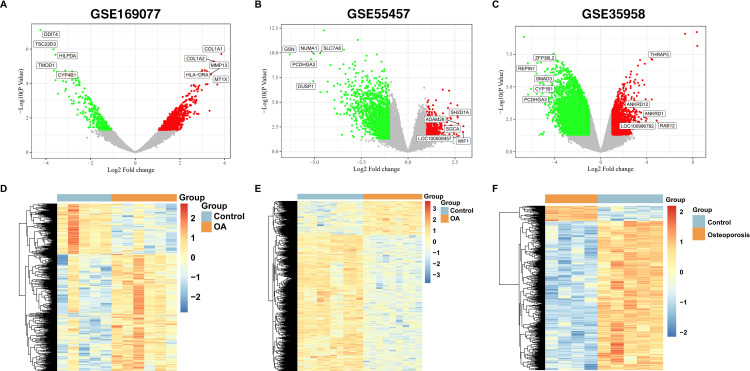
Differential gene expression analysis in OA and OP. The Volcano diagram of Differential expression genes in (A) GSE169077, (B) GSE55457 and (C) GSE35958; The heatmap of differently expressed genes in (D) GSE169077, (E) GSE55457 and (F) GSE35958 datasets across various samples.

In the analysis of the OA group patients from the GSE169077 and GSE55457 datasets, we identified 688 and 370 significantly upregulated genes and 291 and 1246 significantly downregulated genes, respectively. In the GSE35958 dataset, which focused on OP patients, we found 1326 significantly upregulated genes and 5145 significantly downregulated genes (Fig 1).

### Functional enrichment analysis reveals common mechanisms in osteoarthritis and osteoporosis

**Fig 2:**
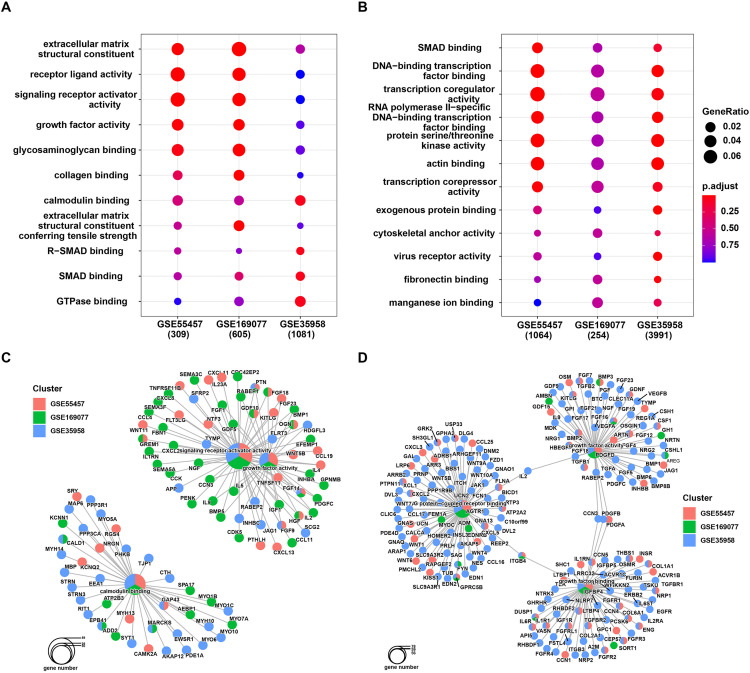
GO Enrichment analysis. (A) GO Enrichment analysis results for up-regulated genes and (B) down-regulated genes; (C) GO network analysis results for down-regulated genes and (D) up-regulated genes.

Performing functional enrichment analysis on the aforementioned differentially expressed genes is beneficial for understanding the biological mechanisms associated with OP and OA. The results of GO enrichment analysis indicate that significantly expressed genes in all three datasets are enriched in several common GO terms, mainly linked to functions related to tissue structural growth and pathways associated with gene expression regulation (Fig 2A and 2B).

Further analysis of shared GO terms reveals that significantly downregulated genes, such as ITGB4 and GDPRC5B, in all three datasets are enriched in G protein-coupled receptor binding functions, while genes like VEGFA and ITGB4 participate in growth factor activity and growth factor binding functions (Fig 2C). Significantly upregulated genes, such as PTN and FGF14, are involved in signaling receptor activator activity and growth factor activity processes, while GAP43, CALD1, and MARCKS are associated with calmodulin binding (Fig 2D). This suggests that the occurrence of OA or OP may be related to intracellular GPCR signaling pathways, calcium-binding proteins, growth factor binding, and activity processes.

**Fig 3:**
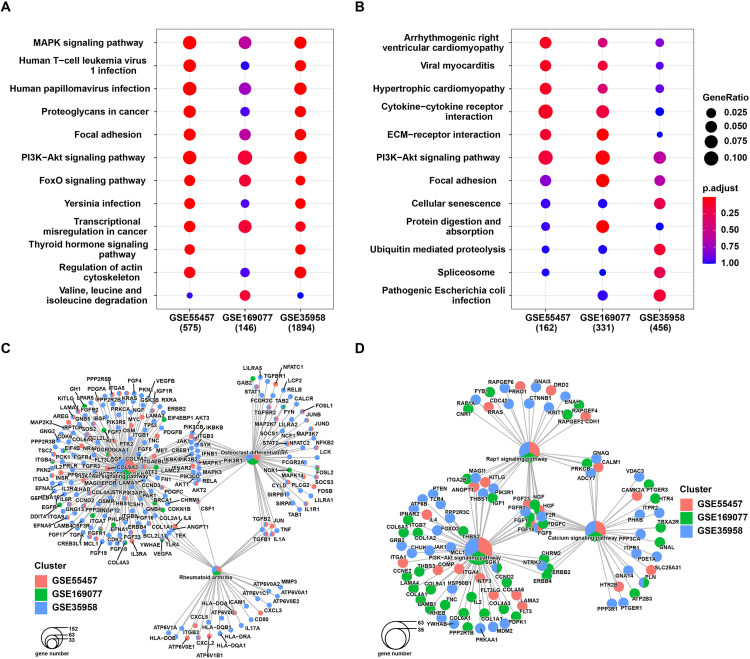
KEGG pathway enrichment analysis. (A)The KEGG pathway enrichment analysis results for up-regulated genes and (B) down-regulated genes; (C) The KEGG network analysis results for down-regulated genes and (D) up-regulated genes.

In our investigation, we conducted KEGG pathway analysis on the differential genes identified in the three datasets, revealing strikingly similar enrichment patterns. Upregulated genes in all datasets displayed a notable preference for pathways such as ECM-receptor interaction, PI3K-Akt, and Cellular senescence (Fig 3A). Conversely, downregulated genes consistently exhibited enrichment in pathways related to PI3K-Akt and FoxO signaling, which play pivotal roles in cellular proliferation, metabolism, and apoptosis (Fig 3B).

Furthermore, by constructing expression-related network diagrams, we discerned a convergence of genes in multiple KEGG pathways. Notably, VEGFA, ITGB4, and FGFR1, which were significantly down-regulated in all three datasets, were discovered to intersect with the PI3K-Akt signaling pathway, a noteworthy finding. Additionally, VEGFA was implicated in the Rheumatoid arthritis process, while FOSL2 was associated with Osteoclast differentiation (Fig 3C). Conversely, upregulated genes collectively converged onto pathways like PI3K-Akt and Calcium signaling (Fig 3D).

### Integrated analysis reveals common genes in OA and OP

**Fig 4:**
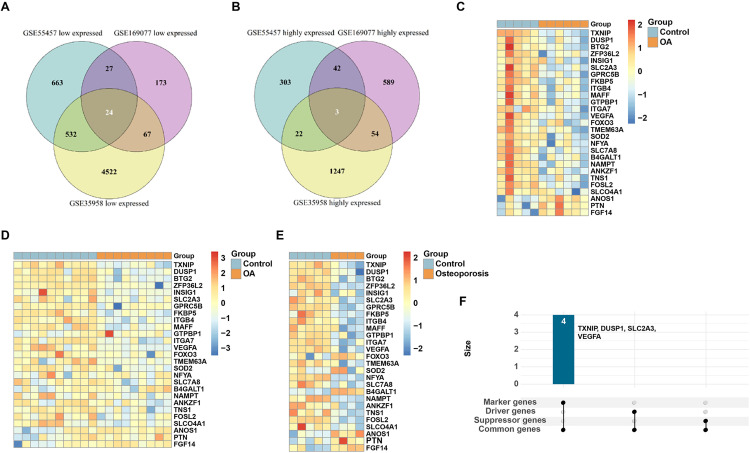
Common genes identification. Venn Diagram of (A) Down-regulated genes and (B) Up-regulated genes; Expression of overlapping genes in datasets (C) GSE169077, (D) GSE55457, and (E) GSE35958; (F) Genes associated with ferroptosis.

Combining the results of the differential analysis and functional analysis mentioned above, it becomes evident that the GO and KEGG enrichment analysis results for DEGs in OA and OP patients show resemblances. This suggests the potential existence of shared mechanisms underlying the onset of these two diseases. Consequently, we further conducted an integrated analysis to identify genes that displayed significant changes across all three datasets. As depicted in Fig 4A and 4B, three genes showed significant upregulation, and twenty-four genes displayed significant downregulation consistently in all three datasets. These overlapping genes were effective in clustering samples within each dataset (Fig 4C-E).

In the context of genes and functional pathways associated with OA or OP, we placed particular emphasis on Ferroptosis-related pathways. To investigate whether ferroptosis is indeed related to the occurrence of OA or OP, we obtained genes associated with ferroptosis from the FerrDb database. Subsequently, we performed integrated analysis with the aforementioned twenty-seven overlapping genes. The results revealed that four of these genes (TXNIP, DUSP1, SLC2A3, and VEGFA) are related to ferroptosis (Fig 4F).

### The ferroptosis levels in BMSCs increase during the progression of osteoporosis

**Fig 5:**
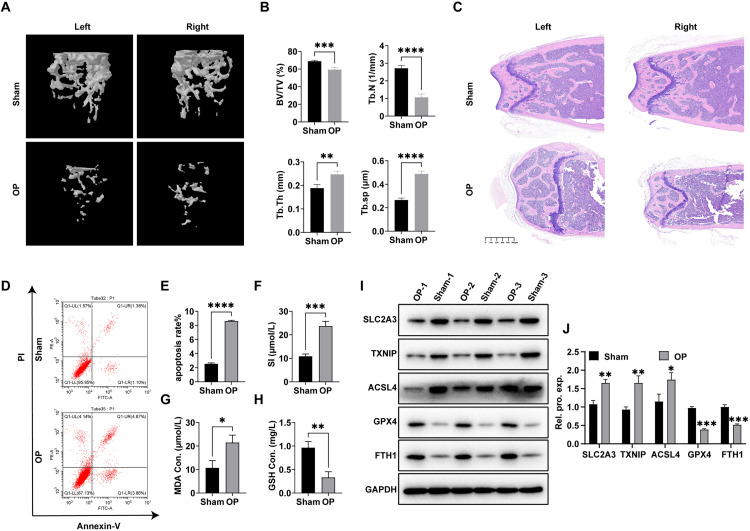
Elevation of ferroptosis levels in BMSCs during OP progression. (A)The micro-CT results of mouse bone tissue and (B) Bone volume over tissue volume (BV/TV), number of trabeculae (Tb.N), bone trabecular thickness (Tb.Th) and trabecular separation (Tb.Sp) were determined. (C) H&E staining results of mouse bone tissue; (D) Flow cytometry analysis of BMSCs apoptosis levels; (E) Statistical analysis of (D); Levels of (F) Fe2+, (G) MDA, and (H) GSH in BMSCs; (H) Levels of SLC2A3, TXNIP, ACSL4, GPX4, and FTH1 in BMSCs; (I) Statistical analysis of (H).

In order to investigate the presence of abnormal ferroptosis (ferroptosis) phenomena in OP patients and the expression of the aforementioned overlapping genes, we employed the OVX modeling method to establish an OP mouse model. The micro-CT results for 3D reconstruction indicated a decrease in trabecular bone quantity and lower density in the experimental group. Furthermore, BV/TV and Tb. N showed significant decrease in the OP model mice, while the value of Tb. Sp was slightly increased (Fig 5A and 5B). In the model group, HE staining revealed a decrease in the quantity of femoral trabeculae and a decrease in bone density (Fig 5C). The combination of these factors confirmed the successful creation of the OP mouse model. After isolating the BMSCs, we discovered a notable rise in cellular apoptosis levels in the BMSCs of the OP mice (Fig 5D and 5E). Additionally, the levels of Fe^2+^ and MDA were significantly elevated in the OP group compared to the control group (Fig 5F and 5G), while the level of GSH was significantly lower (Fig 5H).

Furthermore, we also assessed the levels of SLC2A3, TXNIP, and the ferroptosis markers ACSL4, GPX4, and FTH1 within BMSCs, as depicted in Fig 5I and 5J. These findings are in alignment with the results obtained from sequencing analysis.

## Discussion

Recent research has increasingly spotlighted ferroptosis, a unique form of iron-dependent cell death, as a crucial factor in the pathogenesis of both OA and OP, revealing novel therapeutic targets and mechanistic insights. In OA, ferroptosis has been identified as a significant contributor to chondrocyte death and the degradation of articular cartilage. For instance, Yang et al. [22] and Guo et al. [23] provide compelling evidence that manipulating ferroptotic pathways can mitigate OA progression. Specifically, they point out that the inhibition of ferroptosis in chondrocytes, through agents like deferoxamine, an iron chelator, alleviates inflammation and cartilage erosion, suggesting a protective mechanism against OA.

In the context of OP, ferroptosis influences the viability and function of bone cells, particularly osteoblasts. Studies by Gao et al. [24] and Jiang et al. [25] have demonstrated that iron overload can trigger ferroptosis in osteoblasts, leading to impaired bone formation and increased bone fragility. Furthermore, the therapeutic potential of targeting ferroptosis was underscored by Deng et al. [11], who showed that mangiferin, a natural antioxidant, effectively inhibits osteoblastic ferroptosis via activation of the Nrf2 pathway, thereby enhancing bone density and strength.

We conducted a joint analysis of transcriptomic data from patients with OA and OP, utilizing datasets from the GEO.. Using functional enrichment analyses, we identified significant pathways in the differentially expressed genes, such as PI3K-Akt, cellular senescence, and ECM-receptor interaction, highlighting their involvement.. These pathways are crucial in cell proliferation, metabolism, apoptosis, and extracellular matrix interactions, suggesting a complex interplay in the disease mechanisms of OA and OP. Studies such as Feng et al. emphasize the role of ECM-receptor interactions in BMSC differentiation in OP [26], while Ma et al. discuss the modulation of fatty acid metabolism through the PI3K-Akt pathway in treating OP [27]. Additionally, Pignolo et al. and Jing et al. highlight the impact of cellular senescence on bone health, suggesting therapeutic avenues through senescence management [28,29]. Collectively, these studies underscore the complexity of OP and highlight the therapeutic potential of targeting these pathways.

Furthermore, we used the FerrDb database to identify four genes associated with ferroptosis. The integration of ferroptosis-related genes such as TXNIP, DUSP1, SLC2A3, and VEGFA highlighted their potential role in the pathogenesis of these conditions. Significant expression changes in these genes, consistent across our dataset analyses, could be linked to the cellular damage observed in both diseases. The studies collectively emphasize the regulation and impact of specific ferroptosis-related genes like SLC2A3, TXNIP, ACSL4, GPX4, and FTH1. SLC2A3, identified as a significant player in OP ferroptosis, suggests a role in managing iron homeostasis and oxidative stress within bone cells [30]. TXNIP, which plays a role in reacting to oxidative stress, was discovered as a potential indicator for glucocorticoid-induced osteonecrosis of the femoral head, connecting ferroptosis to the development of this bone disorder [31]. ACSL4 is a crucial enzyme in lipid metabolism that facilitates the execution of ferroptosis. Studies have shown its upregulation in OA, suggesting that modifying ACSL4 expression could mitigate disease progression by influencing lipid peroxidation processes [32]. GPX4 stands out in multiple studies as a central anti-ferroptotic agent that protects cells by reducing lipid peroxides. Its modulation has been linked to reduced cartilage degradation in OA and improved survival rates in models of bone marrow injury, making it a promising target for therapeutic intervention [33]. FTH1, involved in iron storage and regulation, plays a protective role against ferroptosis by regulating iron availability, as demonstrated in bone marrow-derived mesenchymal stem cells [34].

Some findings underscore the importance of managing BMSCs health to mitigate OP. Hu et al. demonstrate that GPX7 is vital for BMSCs osteoblastogenesis, indicating its protective role against osteoporotic deficits by modulating ER stress and the mTOR pathway [35]. Similarly, Jing et al. found that tobacco toxins exacerbate OP through ferroptosis in BMSCs [29], while Meng et al. suggest that Coenzyme Q10 protects against OP by improving mitochondrial function in BMSCs [36]. By establishing an OP mouse model, we confirmed elevated ferroptosis levels in BMSCs, along with a significant decrease in TXNIP and SLC2A3. Our research demonstrates the relevance of ferroptosis to the development of OA and OP. Furthermore, our analysis of gene expression levels has enhanced our comprehension of the mechanisms involved in these conditions.

The strengths of our study include the use of integrated transcriptomic data to identify potential pathogenic genes and the validation of these findings through well-established in vivo models and molecular techniques. The study’s limitations are primarily centered on its exploratory scope and partial coverage of ferroptosis in disease contexts. While comprehensive in integrating transcriptomic data to identify pathogenic genes, the research predominantly focuses on OP, with less extensive examination of OA. This disparity highlights a potential gap in fully understanding ferroptosis’s role across both conditions. Future research should extend these findings through experimental models to confirm ferroptosis’s involvement in OA, potentially enriching the therapeutic landscape for musculoskeletal diseases.. Future research could aim to validate the involvement of ferroptosis in OA through similar experimental approaches, potentially providing a more comprehensive understanding of its role in musculoskeletal diseases.

Moreover, our research could delve deeper into the specific pathways and interactions between the identified ferroptosis-related genes. For example, exploring the direct interactions and regulatory mechanisms of these genes could uncover new therapeutic targets or biomarkers for OA and OP. Additionally, the differential impact of these pathways in various cell types within the bone microenvironment remains to be elucidated, which could reveal cell-specific therapeutic strategies.

Overall, our research enhances the comprehension of the molecular pathways involved in OA and OP, emphasizing the importance of ferroptosis. The identified pathways and genes offer potential new avenues for therapeutic intervention and diagnostic advancements. However, the necessity for further exploration and validation in different models and additional molecular studies remains imperative to fully harness these insights for clinical application.

## Conclusion

The research we conducted highlights the important impact of ferroptosis on the development of OP and OA. By integrating transcriptomic data and in vivo models, we identified key ferroptosis-related genes and pathways that contribute to both conditions, offering novel insights for therapeutic interventions.

## Supporting information

S1 Fig PCA analysis. PCA analysis of the datasets of (A) GSE169077, (B) GSE35958 and (C-D) GSE55457.
(TIF)

## Abbreviations

OA: Osteoarthritis
OP: Osteoporosis
GEO: Gene Expression Omnibus
DEGs: Differentially Expressed Genes
BMSCs: Bone Marrow Stromal Cells
PI3K-Akt: Phosphoinositide 3-kinases-Protein Kinase B
ECM: Extracellular Matrix
FOXO: Forkhead Box O
GPCR: G Protein-Coupled Receptor
MDA: Malondialdehyde
GSH: Glutathione
CT: Computed Tomography
H&E: Hematoxylin and Eosin
MSA: Multiple Sequence Alignment
TBST: Tris-Buffered Saline and Tween 20
HRP: Horseradish Peroxidase
Nrf2: Nuclear Factor Erythroid 2-Related Factor 2
