# Supplementary figures and images for: Multimodal analysis investigating the shared pathogenic mechanisms of osteoporosis and osteoarthritis with an initial exploration of the role of ferroptosis
Source: PLoS One. 2025 Sep 25;20(9):e0332769. doi: 10.1371/journal.pone.0332769 (PMC12463252; doi:10.1371/journal.pone.0332769)

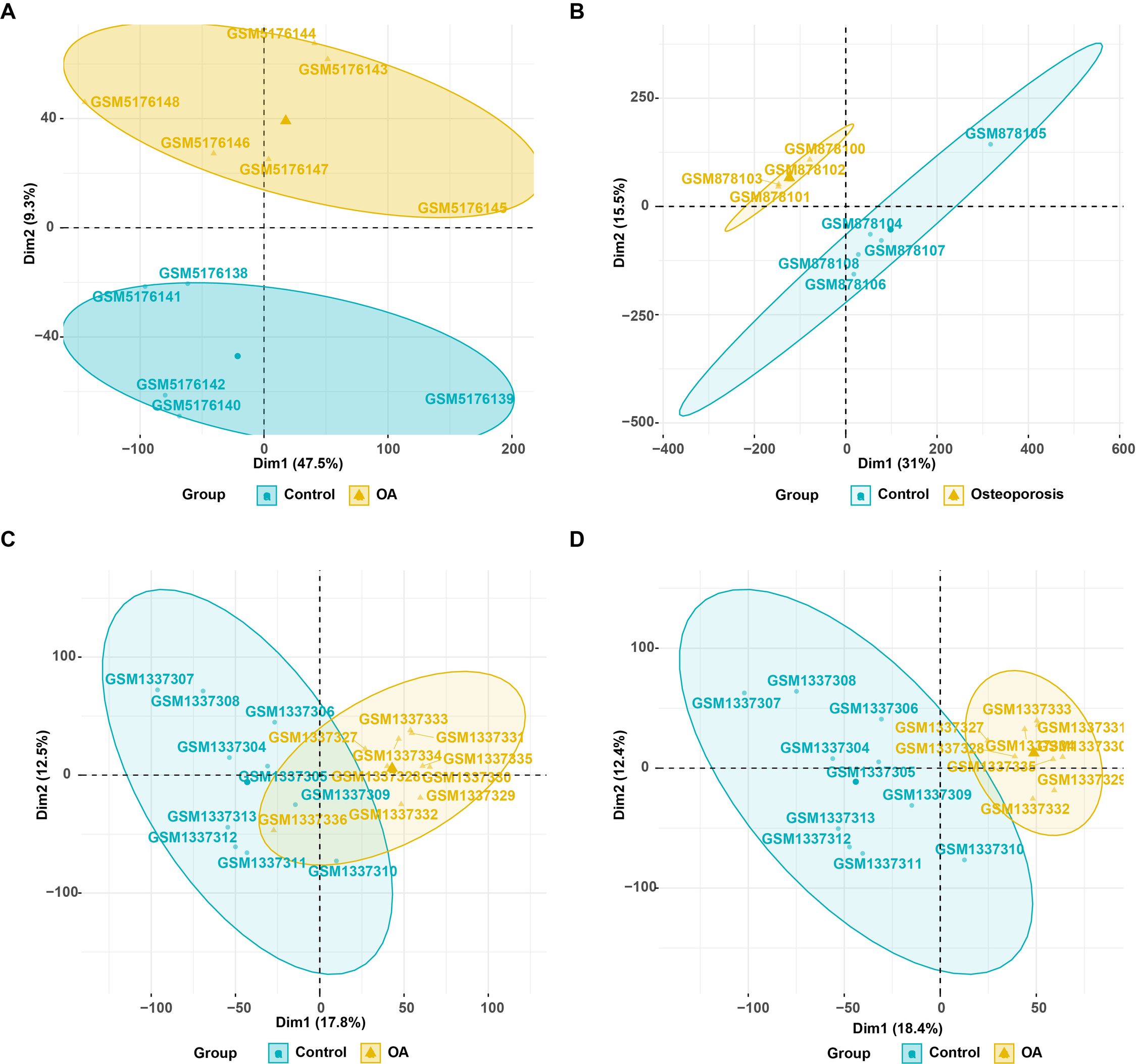

Supplement: S1 Fig — PCA analysis of the datasets of (A) GSE169077, (B) GSE35958 and (C-D) GSE55457. (TIF) [file pone.0332769.s001.tif]
